# Triple-Band and Ultra-Broadband Switchable Terahertz Meta-Material Absorbers Based on the Hybrid Structures of Vanadium Dioxide and Metallic Patterned Resonators

**DOI:** 10.3390/ma16134719

**Published:** 2023-06-29

**Authors:** Yuke Zou, Hongyan Lin, Gaowen Tian, Haiquan Zhou, Huaxin Zhu, Han Xiong, Ben-Xin Wang

**Affiliations:** 1 School of Science, Jiangnan University, Wuxi 214122, China; 2 Zhejiang Beyondsun Green Energy Technology Co., Ltd., Huzhou 313008, China; 3 School of Electrical Engineering, Chongqing University, Chongqing 400044, China

**Keywords:** terahertz meta-materials, bifunctional absorber, triple-band and ultra-broadband absorption, vanadium dioxide

## Abstract

A bifunctional terahertz meta-material absorber with three layers is designed. The surface of the bifunctional meta-material absorber is a periodically patterned array composed of hybrid structures of vanadium dioxide (VO_2_) and metallic resonators; the middle layer is a nondestructive TOPAS film, and the bottom layer is a continuous metallic plane. Utilizing the phase-transition property of VO_2_, the responses of the meta-material absorber could be dynamically switched between triple-band absorption and ultra-broadband absorption. When VO_2_ is in the metallic state, an ultra-broadband absorption covering the bandwidth of 6.62 THz is achieved over the range from 4.71 THz to 11.33 THz. When VO_2_ is in the di-electric state, three absorption peaks resonated at 10.57 THz, 12.68 THz, and 13.91 THz. The physical mechanisms of the bifunctional meta-material absorber were explored by analyzing their near-field distributions. The effects of varying structural parameters on triple-band and ultra-broadband absorption were investigated. It is revealed that by optimizing the structure parameters, the number of absorption peaks could be increased for a certain sacrifice of absorption bandwidth. FDTD Solutions and CST Microwave Studio were used to simulate the data of the absorber, and similar results were obtained.

## 1. Introduction

A THz region does not have strict borders; however, usually, the range of 0.1 to 100 THz is considered to be the correct region. This has unique advantages, such as strong penetrating power, low energy, and good transient performance [1,2], and has broad application prospects in many fields in terms of high-speed wireless communication [3,4], material detection [5], biomedical research [6,7], and nondestructive evaluation [8]. In recent years, with the development of technology, breakthroughs have been made in the research of stable broadband terahertz sources [9,10]. Although the research on terahertz devices has received extensive attention, it is difficult for terahertz waves to interact strongly with natural materials, resulting in great resistance to their development [11,12]. Meta-materials seem to provide an effective way to solve the problem of the weak interaction between the terahertz waves and matters.

Meta-material, a kind of artificial composite material with periodic units, owns many exotic properties that do not exist in nature, such as the anomalous Doppler effect, negative refractive index, etc. These effects mainly come from their unit structures rather than the substances making them up [13]. As we all know, photonic crystals have multiple films (the refractive index of the films varies periodically in space [14]), which achieve interference cancellation through multiple reflections. In this case, certain frequency bands of light will be canceled out, and the selective transmission (reflection) of light is achieved. If a surface with microstructures is used, it is possible to reduce the number of layers of artificial materials while maintaining interference cancellation and achieving thinness and lightness. Of course, there are also reports introducing meta-materials into photonic crystals to obtain better performance [15] or oxide thin films to obtain tunable properties [16].

In 2008, Landy et al. presented the first meta-material absorber with near-perfect absorption in the microwave range [17]. Its thickness is only one-twenty-fifth of the resonance wavelength, which lays a solid foundation for the development of lightweight and ultra-thin absorbing devices [18]. In 2011, J. Grant et al. reported a polarization-insensitive resonant meta-material absorber and carried out simulations, experiments, and measurements on it in the terahertz band [19]. This absorber has a classic three-layer structure of “metal-dielectric-metal” with periodically distributed ring-shaped cross resonators. In the same year, Yong Ma et al. reported a dual-band meta-material absorber [20]. They simulated the absorption curve of the absorber by the finite-difference time-domain method and characterized the manufactured samples by measuring the reflectance spectrum. In 2014, Yin Zhang et al. reported a graphene-based tunable meta-material absorber and explained the tuning by the change in the effective inductance of the graphene wire under gate voltage bias [21].

Since then, various meta-materials [22,23,24], the resonance functions of which include absorption [25], filtering [26], electromagnetic induction transparency [27], and polarization conversion [28], have been widely proposed. However, these meta-materials generally have fixed resonance responses; that is, their optical properties cannot be actively adjusted. At present, a large number of proposed terahertz meta-materials are mainly based on the interaction of their metallic resonators with incident light [29,30,31]. A widely used design scheme for tunable meta-materials is to replace metallic substances in traditional meta-materials with substances for which electrical conductivity can be affected by external environmental parameters. In this way, the conductivity of resonators can be changed by changing the environmental parameters, and the functions of the meta-materials can be controlled.

In terms of material selection, graphene [32,33], photosensitive silicon [34,35], and vanadium dioxide [36,37] have received extensive attention. If the Fermi level is changed by applying a voltage, the conductivity of graphene could be varied. However, it is necessary to lay external electrodes on the graphene layer to change the Fermi level, which increases the complexity of design and production. In addition, graphene’s ultra-thin feature poses challenges to manufacturing. By varying the intensity of the pump light incident on the photosensitive silicon, the conductivity of the photosensitive silicon can also be changed. However, the conductivity tuning range of photosensitive silicon is not great, which makes it difficult to achieve a large modulation depth for tunable meta-materials made by photosensitive silicon. In addition, to realize conductivity modulation, the pumping light incident on the photosensitive silicon usually has great light intensity. In this case, it is difficult to ensure that the meta-material has a long lifetime.

Phase-change VO_2_ is an available material, and it can be switched between the metallic and di-electric states with temperature [38]. The state change of VO_2_ is mainly reflected in a change in the conductivity. The higher the temperature is, the higher the conductivity is, and VO_2_ has phase transition temperature points. When the temperature of VO_2_ is far away from the phase transition point, the conductivity changes slightly with the change in temperature. When the temperature is close to the phase transition point, the conductivity of VO_2_ changes drastically with temperature. When passing through the phase transition points, its conductivity can be changed by several orders of magnitude. Due to the thermal hysteresis effect, when the temperature rises and falls, the phase transition points of VO_2_ are different: about 340 K and 330 K, respectively [39,40,41].

Although a large number of tunable terahertz absorbers using phase-change materials, such as VO_2_, have been proposed, most of them only provide the ability to adjust the resonance frequency or absorption intensity [42,43,44,45,46,47,48]. Only some meta-material absorbers are designed to achieve the switching between broadband absorption and multiband absorption. There is a simple idea to realize this function [49,50,51,52,53]: resonators made from phase-change materials and metallic resonators. When the phase-change material is in the di-electric state, only the metallic resonators can resonate with the incident wave. When the phase-change material is in the metallic state, all resonators can respond to the incident waves. In this way, the absorber has different resonance modes with the phase-change material in different states, and it is possible to realize the switching between broadband absorption and multiband absorption. Based on this, some terahertz absorbers have been reported, as shown in Table 1 [49,50,51,52,53,54,55,56]. Some absorbers have two absorption modes, but their absorption bandwidth is too small. Some absorbers have a large absorption bandwidth but do not have a multiband absorption mode. The absorber proposed in this article has both advantages.

In this work, a bifunctional terahertz meta-material absorber is proposed. It has a classic three-layer structure. From top to bottom, they are periodic arrays composed of patterned gold resonators and VO_2_ resonators and TOPAS di-electric layer and gold plane, respectively. The bifunctional absorber can achieve reversible conversion between triple-band absorption and ultra-wideband absorption in the terahertz band. When VO_2_ is in the metallic state, an ultra-broadband absorption bandwidth of 6.62 THz is achieved over the range from 4.71 THz to 11.33 THz. When VO_2_ is in the di-electric state, only gold resonators participate in response to the incident light. The absorption responses of three frequency bands are realized, and the frequencies at 10.57 THz, 12.68 THz, and 13.91 THz have absorption intensities of 96.9%, 81.9%, and 91.9%, respectively. The physical mechanisms of ultra-broadband absorption and triple-band absorption are discussed by analyzing their near-field distributions. The influence of structure parameters on the bifunctional resonance responses is also discussed.

The designed bifunctional meta-material has great application potential in the terahertz frequency band. First, the designed absorber has the potential to be processed into a photocatalyst [57]. The ultra-wideband absorption gives the meta-material a high utilization rate for terahertz waves, and the surface plasmon resonance caused by the incident wave can provide a stable surface current. The persistent photogenerated charges on the meta-surface are beneficial to promoting various redox mechanisms. The meta-material can be further processed into a photocatalyst by a solvothermal method, ultrasonic-assisted and microwave-assisted methods, thermal decomposition methods, and so on [57]. Second, the designed meta-material can be applied in photodetection [58]. For photodetectors, sufficient light absorption is the main prerequisite for efficient photocurrent conversion. Therefore, the absorber can then have an optical response to terahertz waves over a wide frequency range or at frequency points. Third, the designed meta-material can be applied to electromagnetic stealth. In order to achieve electromagnetic stealth, high-performance electromagnetic absorbers can be used to attenuate electromagnetic echoes and convert them into thermal energy [59]. Moreover, the absorber proposed in this paper has the characteristics of being light and thin and can adapt to complex external environments.

When compared to previous articles [49,50,51,52,53,54,55,56], this paper has the following novelties. First, the structure is simple. Many articles propose meta-material absorbers with multiple layers patterned to achieve ultra-wideband absorption. This undoubtedly increases the structural complexity. The proposed meta-material in this article consists of only three layers, with only one film being patterned. Second, we use two kinds of software to simulate the proposed model. The simulation results of the two software have a high similarity, which indicates the reliability of the results. Third, the absorber proposed in this paper has both the advantages of dual functionality and a large bandwidth. Many absorbers only have one of the two advantages, with ultra-broadband absorption or switching functionality between broadband absorption and multiband absorption.

## 2. Materials and Methods

**Figure 1:**
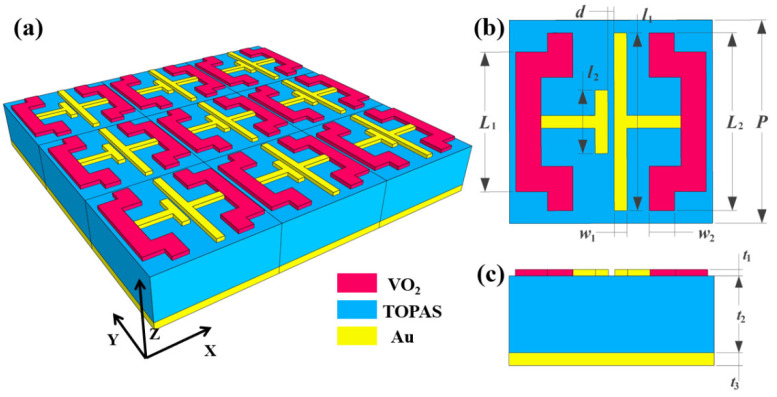
(**a**) Schematic diagram of the three-dimensional structure of the absorber. (**b**) Top view of the absorber. (**c**) Side view of the absorber.

The schematic diagram of the designed bifunctional meta-material absorber is shown in Figure 1. Excluding the substrate supporting the structure, the bifunctional meta-material absorber consists of three core layers. The bottom layer is a gold plate with a thickness greater than the skin depth of the terahertz band, guaranteeing near-zero transmittance throughout the meta-material absorber [60]. The middle layer is a lossless di-electric material, TOPAS, with a di-electric constant of 2.35 [61]. The top layer is a periodic patterned array composed of VO_2_ resonators and gold resonators, dividable into two gold resonators in the middle and two VO_2_ resonators on both sides for a strong response to the incident waves.

According to energy conservation, the normalized incident light wave irradiates the meta-material absorber with the following results: *R*(*ɷ*) + *T*(*ɷ*) + *A*(*ɷ*) = 1. Here, *R*(*ɷ*) is the reflectance, *T*(*ɷ*) is the transmittance, and *A*(*ɷ*) is the absorption. Due to the presence of the bottom gold plane, the transmission of the designed meta-material structure is close to zero. At this point, the absorption can be expressed as *A*(*ɷ*) = 1 − *R*(*ɷ*). Once the structure parameters of the meta-material absorber are controlled so that the effective impedance of the entire absorber is close to the free space impedance (377 Ω), the near-zero reflection of the absorber can be achieved; that is, high absorption could be realized. The impedance matching theory can be expressed as [62,63,64]:

(1) $$A\left(\omega \right)=1-R\left(\omega \right)=1-{\left(\frac{Z-Z_{0}}{Z+Z_{0}}\right)}^{2}=1-{\left(\frac{Z_{r}-1}{Z_{r}+1}\right)}^{2}$$

(2) $$Z_{r}=\sqrt{\frac{{\left[1+S_{11}\left(\omega \right)\right]}^{2}-S_{21}{\left(\omega \right)}^{2}}{{\left[1-S_{11}\left(\omega \right)\right]}^{2}-S_{21}{\left(\omega \right)}^{2}}}$$

here, *Z*_0_ is the effective impedance of free space, and $Z=\sqrt{\mu /\varepsilon }$ is the effective impedance of the designed meta-material absorber. μ is effective permeability, and ε denotes the effective permittivity of the meta-material. *Z*_r_ = *Z*/*Z*_0_ is the normalized impedance between the purposed structure and free space. Through design and optimization, the structural parameters of the dual-function absorber are obtained, as shown in Table 2.

Lumerical FDTD Solutions, which is based on the finite-difference time-domain method, was used to simulate the absorption effect of the designed bifunctional terahertz meta-material absorber. A plane wave is located directly above the absorber and emits the terahertz plane wave along the negative direction of the Z axis. Periodic boundary conditions are set in the X and Y directions to model the periodic array and perfectly matched conditions are set in the Z direction. VO_2_ in the terahertz region can be described by the Drude model [65,66,67,68,69,70]:

(3) $$\varepsilon \left(\omega \right)=\varepsilon_{\infty }-\frac{\omega_{p}^{2}\left(\sigma \right)}{\omega^{2}+i\gamma \omega }$$

(4) $$\omega_{p}^{2}\left(\sigma \right)=\frac{\sigma }{\sigma_{0}}\omega_{p}^{2}\left(\sigma_{0}\right)$$

In Equation (3), $\varepsilon_{\infty }$ = 12 is the di-electric constant at infinite frequency, *ω_p_*^2^(*σ*) is the plasma frequency, and *γ* = 5.75 × 10^13^ rad/s is the collision frequency. In Equation (4), *σ* = 3 × 10^5^ S/m, and the initial value of the plasma frequency is *ω_p_*(*σ*_0_) = 1.4 × 10^15^ rad/s. *σ* = 2 × 10^5^ S/m is set to the metallic state of VO_2_, and *σ* = 2 × 10^2^ S/m is set to the insulating state of VO_2_. When the temperature rises, the internal components of VO_2_ do not undergo phase transition at the same time. At this time, VO_2_ exists in a state of coexistence between the metallic component and the di-electric component. The volume fraction of the metallic component of VO_2_ can be expressed as [40,71]

(5) $$\varphi \left(T\right)=0.95\left\{1-\frac{1}{1+exp\left[\left(T-68\right)/2\right]}\right\}$$

The change in the volume fraction of the components in the metallic state will affect the di-electric function of VO_2_ (*ɛ_c_*), and thus affect its conductivity:

(6) $$\sigma =-i\varepsilon_{0}\omega \left(\varepsilon_{c}-1\right)$$

**Figure 2:**
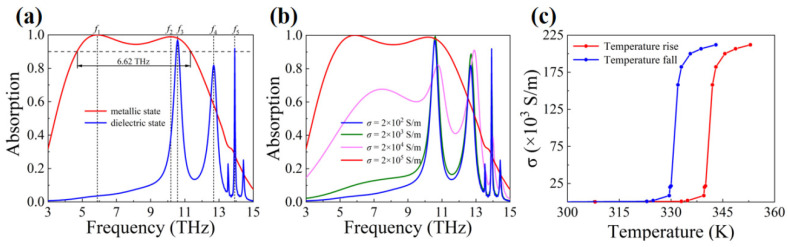
(**a**) Absorption spectrum of the absorber when VO_2_ is in the metallic state and in the di-electric state. (**b**) Absorption spectrum of the absorber with different VO_2_ conductivity. (**c**) Curve of the conductivity of VO_2_ as a function of temperature.

We can use devices, such as dry constant temperature incubators, water baths, and electric heating jackets, to control the temperature [72,73]. Rather than the characteristics of the absorber in the transitional state of VO_2_, we are more concerned with the two absorption modes of the absorber (VO_2_ is completely in the di-electric state or in the metal state). As shown in Figure 2c, when the temperature of VO_2_ is far away from the phase transition point, the change in the temperature will hardly affect the conductivity of VO_2_. Therefore, although the high absorption of the absorber may heat the absorber, it is enough to limit the temperature fluctuations within a certain range by using the above-mentioned temperature control devices. It is worth noting that the phase transition curves of VO_2_ are different when the temperature rises and falls due to the thermal hysteresis effect. However, when the temperature is far away from the phase-transition point, the changes in both curves tend to be flat.

## 3. Results and Discussion

Figure 2a shows the absorption spectra of the designed meta-material absorber. When VO_2_ is in the metal state, an ultra-broadband absorption is realized, and the absorption bandwidth with an absorbance of greater than 90% reaches 6.62 THz over the range from 4.71 THz to 11.33 THz. Two absorption peaks appear in the red absorption curve, having the frequencies of *f*_1_ = 5.88 THz and *f*_2_ = 10.18 THz, and have absorption rates of 99.9% and 98.8%. Moreover, its relative absorption bandwidth (RAB) is 82.5%. The RAB can be expressed as RAB = 2 (*f*_H_ − *f*_L_)/(*f*_H_ + *f*_L_). Here, *f*_H_ and *f*_L_ are, respectively, the maximum and the minimum frequency at an absorption of greater than 90%. When VO_2_ is in the di-electric state, three absorption peaks are realized; the absorption peaks possess the resonance frequencies of *f*_3_ = 10.57 THz, *f*_4_ = 12.68 THz, and *f*_5_ = 13.91 THz, and their absorption rates are 96.9%, 81.9%, and 91.9%, respectively. Figure 2b shows the absorption spectra of the bifunctional meta-material absorber under different VO_2_ conductivity. When the conductivity of VO_2_ is 2 × 10^5^ S/m (metallic state), it can be seen that ultra-broadband absorption is realized. As the conductivity of VO_2_ decreases, the absorption at middle and low frequencies gradually attenuates, while discrete absorption peaks at high frequencies begin to appear and absorption increases.

**Figure 3:**
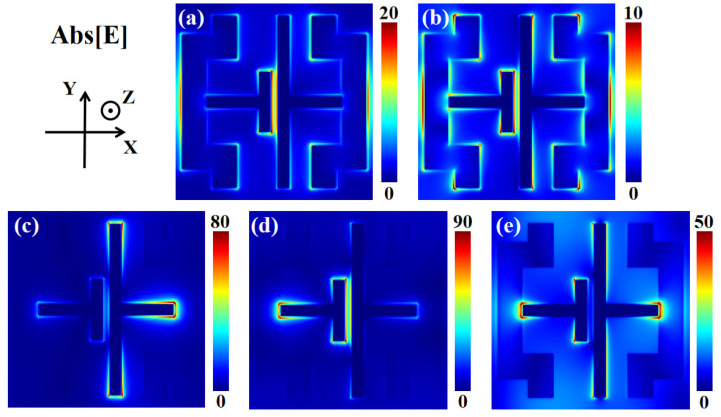
The electric field distribution (Abs [E]) of the absorber at the absorption peaks when VO_2_ is in the metallic state (top) and di-electric state (bottom). (**a**) *f*_1_ = 5.88 THz, (**b**) *f*_2_ = 10.18 THz, (**c**) *f*_3_ = 10.57 THz, (**d**) *f*_4_ = 12.68 THz, and (**e**) *f*_5_ = 13.91 THz.

Figure 3 presents the electric field distributions of the meta-material absorber at each absorption peak. For peak *f*_1_ in Figure 3a, the electric field is mainly concentrated between the two gold resonators and on both sides of the unit cell. This suggests that the absorption at *f*_1_ comes from the coupling effect between the two gold resonators and the coupling effect between the unit cells in the X direction. In Figure 3b, the electric field is not only concentrated between the two gold resonators and on both sides of the unit cell but also around the resonators. This illustrates that there is a complex response mode at *f*_2_, with various parts of the top layer pattern contributing to high absorption. When the external optical field interacts with the metal surface, the energy is transferred to free electrons and surface plasmon waves are generated on the metal surface. When the frequency of the incident wave matches the frequency of the surface plasmon wave, the surface plasmon wave absorbs most of the energy of the incident light. This phenomenon is called surface plasmon resonance (SPR). If the distribution of SPR is very inhomogeneous, it is called localized surface plasmon resonance (LSPR). If the distribution of SPR is relatively uniform, it is called propagating surface plasmon resonance (PSPR). Figure 3c–e show the electric field distributions of the absorber at three absorption peaks when VO_2_ is in the di-electric state. At the frequency of *f*_3_, the electric field is concentrated at the edges of the right gold resonator. It indicates that LSPR is generated near the right gold resonator, and the absorption of the absorber at *f*_3_ is mainly contributed to by the right gold resonator.

At *f*_4_, the electric field energy is concentrated at the edges of the left gold resonator and between the two gold resonators. It indicates that the absorption at the frequency of *f*_4_ comes from the left gold resonator’s own response to the incident waves and the coupling between the two gold resonators. In addition, LSPR is generated here as well. At *f*_5_, the electric field energy is concentrated at the edges of the two gold resonators. The energy density at both the left end of the left gold resonator and the right end of the right gold resonator is large. Besides, there is relatively uniform energy distribution in the area away from the resonator. This indicates that the absorption at *f*_5_ of the meta-material absorber is formed by the co-superposition of the LSPR at the edge of the gold resonator and the PSPR in the unit cell. It is worth noting that there is little electric field energy gathered in the part where the right gold resonator faces the left gold resonator at *f*_3_ and *f*_5_. This may be because the presence of the left gold resonator interferes with the response of the right gold resonator, and this conclusion will be corroborated in the following text (Figure 6). At the frequencies of *f*_3_, *f*_4_, and *f*_5_, the VO_2_ resonators barely respond. This is because these three frequencies describe the absorption peaks with VO_2_ in the di-electric state, and VO_2_ in the di-electric state does not have the ability to respond to the incident waves like metallic resonators. We can find from Figure 3c–e that there is little electric field energy near the VO_2_ resonators.

**Figure 4:**
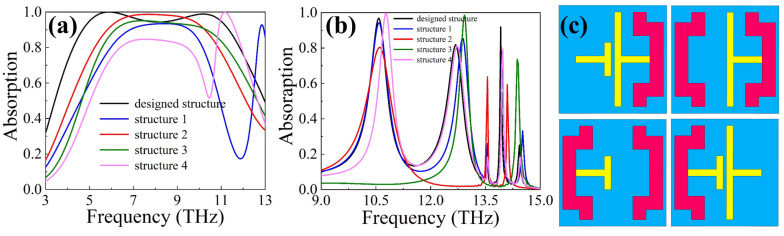
Absorption spectrum of the absorber when one of the resonators is absent. (**a**) Spectrogram when VO_2_ is in the metallic state. (**b**) Spectrogram with VO_2_ in the di-electric state. (**c**) Top view of structures 1–4 (from left to right and from top to bottom).

In order to further explore the response of each resonator to the incident waves, the absorption spectrum of the absorber when one resonator is absent was plotted, as shown in Figure 4. Structures 2 and 3 are the structures of the original absorber without one of the gold resonators. When VO_2_ is in the metallic state, the absorption at both *f*_1_ and *f*_2_ is weakened because the absence of one gold resonator breaks the original coupling between the two gold resonators, as shown in Figure 3a,b. With VO_2_ in the di-electric state, the absorption peak of structure 2 at *f*_2_ disappears, and the absorption peak of structure 3 at *f*_1_ disappears. The absorber’s absorption at *f*_3_ and *f*_4_ comes from the responses of the right and the left gold resonators, respectively, as shown in Figure 3c,d. The absence of the resonators causes the corresponding absorption peaks to disappear. In addition, due to the response of both gold resonators at *f*_5_, the absorptivity of these two structures drops at *f*_5_.

Structures 1 and 4 are the structures of the original absorber without one of the VO_2_ resonators. It can be seen that when VO_2_ is in the metallic state, the absorptions of the two structures at *f*_1_ and *f*_2_ both attenuate, but a new absorption peak is generated in the high-frequency band, respectively. This is because the absence of one of the VO_2_ resonators disrupts the original intercellular coupling mode and creates a new response mode. From Figure 3a,b, it can be seen that the left and right boundaries of the unit cell have a strong resonance, which plays an important role in absorption. Taking structure 1 as an example, when the left VO_2_ resonator is missing, the left gold resonator is exposed to the left adjacent unit cell. A new coupling between the left gold resonator in this unit and the right VO_2_ resonator in the left adjacent unit cell is generated. The effect of structure 4 is similar. When VO_2_ is in the di-electric state, the absorption rates of the two structures do not change much because the VO_2_ resonators in the di-electric state respond weakly to the incident waves, as shown in Figure 3c–e.

**Figure 5:**
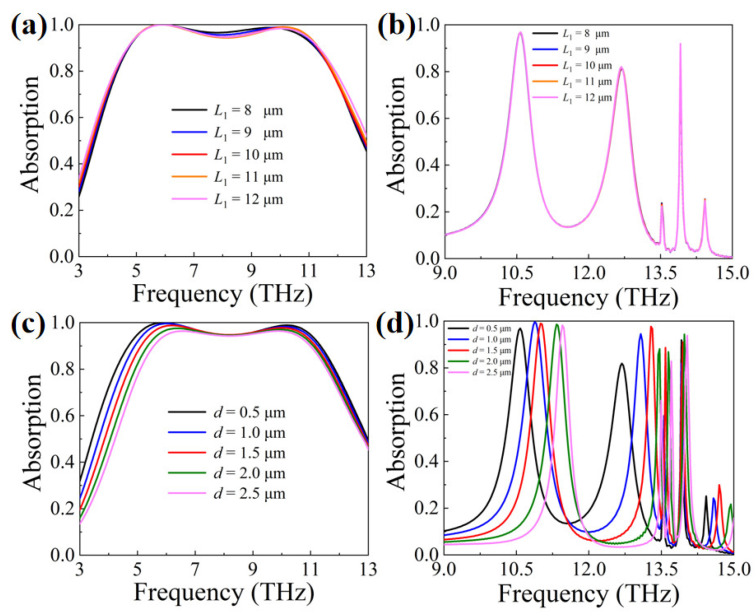
Effect of structural parameters on absorption when VO_2_ is in the metallic or in the di-electric state. (**a**) *L*_1_, metallic state; (**b**) *L*_1_, di-electric state; (**c**) *d*, metallic state; (**d**) *d*, di-electric state.

Figure 5a,b show the effect of *L*_1_ on the absorption effect. When VO_2_ is in the metallic state, the change in *L*_1_ has little effect on the absorption response. It can be seen from Figure 3a that the electric field energy at the edges of the unit cell only covers part of the VO_2_ resonators, which makes the change in *L*_1_ have little effect on the coupling effect between the unit cells. When VO_2_ is in the di-electric state, the absorption rate is almost unchanged because the VO_2_ resonators no longer participate in response to the incident light. Figure 5c,d demonstrate the effect of the structure parameter *d* (the distance between two metallic resonators in the unit cell) on absorption. When VO_2_ is in the metallic state, the absorption at *f*_1_ decays gradually as *d* increases, while the absorption at *f*_2_ changes little as *d* changes.

The reason can be found in Figure 3a,b, and their descriptions. At the frequency of *f*_1_, absorption is only contributed to by the coupling between the two gold resonators and the coupling between the unit cells. At *f*_2_, besides the two coupling modes described above, there are four resonators that have their own responses to the incident waves. Therefore, high absorption has a stronger dependence on the coupling effect between the two gold resonators at *f*_1_; that is, the value of *d* has a greater influence on absorption at *f*_1_. When VO_2_ is in the di-electric state, as *d* increases, the three absorption peaks are all blue-shifted due to the LSPR effect. The LSPR effect can be explained as [30,74]

(7) $$\lambda_{\mathrm{LSPR}}=2\pi c\sqrt{\frac{LC}{2}}=2\pi c\sqrt{\frac{\left(L_{m}+L_{e}\right)\left(C_{m}+C_{e}\right)}{2}}$$

here, *L_m_* and *L_e_* are the inductance between the resonator and the metallic substrate and the inductance between adjacent resonators, respectively. *C_m_* and *C_e_* are the capacitance between the resonator and the metallic substrate and the capacitance between adjacent resonators, respectively. When *d* increases, the distance between the two gold resonators increases (*C_e_* decreases), leading to a decrease in *λ*_LSPR_ and, thus, a blue shift of the absorption peak. It is worth noting that the blue shift of the absorption peak at *f*_5_ is significantly slower than the absorption peaks at *f*_3_ and *f*_4_. This is because the absorption at *f*_5_ originates from both LSPR and PSPR, while the absorption at *f*_3_ and *f*_4_ is only contributed to by LSPR, which leads to a weaker influence of the LSPR effect at *f*_5_.

**Figure 6:**
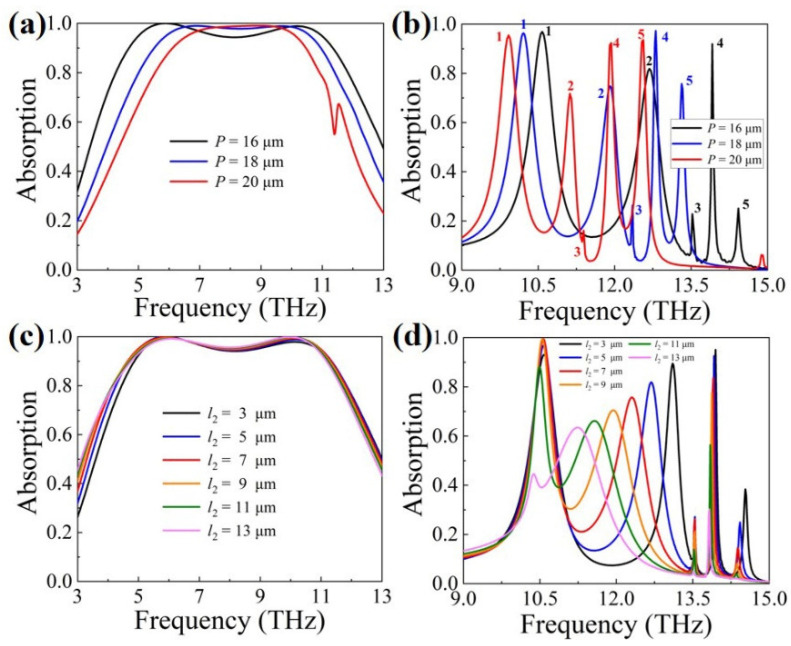
Effect of structural parameters on absorption when VO_2_ is in the metallic or di-electric state. (**a**) *P*, metallic state; (**b**) *P*, di-electric state; (**c**) *l*_2_, metallic state; (**d**) *l*_2_, di-electric state.

Figure 6a demonstrates the effect of period *P* on absorption when VO_2_ is in the metallic state. It can be seen from the figure that as *P* increases, the absorption bandwidth decreases gradually because at *f*_1_ and *f*_2_, the coupling between the unit cells greatly contributes to the absorption of the absorber, as shown in Figure 3a,b. Figure 6b is the spectrogram about the structure parameter *P* when VO_2_ is in the di-electric state. All three curves have five absorption peaks in the studied band (including the absorption peaks with a low absorption rate), and they are marked with numbers 1–5, respectively. With an increase in *P*, the five absorption peaks are all red-shifted. To some extent, the increase in *P* is equivalent to the amplification of the structure in the direction perpendicular to the propagation direction, and the matching condition between the incident light and the lateral waveguide mode will be satisfied at longer wavelengths, resulting in the red shift of the resonance wavelength [75]. It is worth noting that the absorption rate of absorption peak 5 increases dramatically with an increase in period *P*. This shows that under the premise of sacrificing a certain bandwidth in the broadband absorption, the absorber has the potential to increase the number of absorption peaks with sufficiently large absorptivity in the multiband absorption mode.

Figure 6c,d show the effect of the value of *l*_2_ on absorption when VO_2_ is in the metallic state and di-electric state, respectively. When VO_2_ is in the metallic state, ultra-broadband absorption is not damaged because the increase in *l*_2_ hardly affects the coupling between the two gold resonators. When VO_2_ is in the di-electric state, the absorption intensity at *f*_3_ and *f*_5_ gradually weakens, and the absorption peak at *f*_4_ red shifts. At the frequencies of *f*_3_ and *f*_5_, the increase in the length of the left gold resonator (*l*_2_) gradually interferes with the response of the right gold resonator to the incident waves, resulting in the attenuation of the absorption. This effect is the same as that shown in Figure 3c,e. The red shift at *f*_4_ can be explained by Equation (7). As the length of the left gold resonator (*l*_2_) increases, the facing area of the two gold resonators increases. It leads to an increase in the capacitance between the two resonators (*C*_e_), which, in turn, red shifts the absorption peak at *f*_4_.

**Figure 7:**
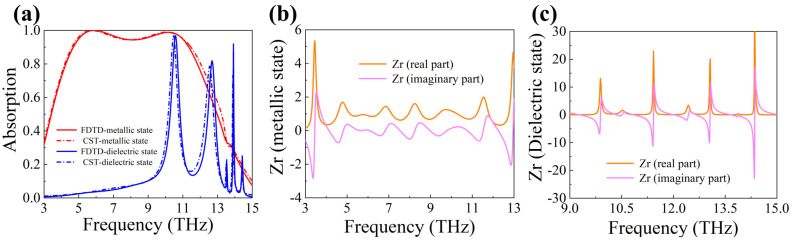
(**a**) Absorption spectra simulated by CST Microwave Studio and FDTD Solutions. (**b**) Relative impedance of the absorber when VO_2_ is in the metallic state. (**c**) Relative impedance of the absorber when VO_2_ is in the di-electric state.

In order to make the results more convincing, the absorption curves of the proposed meta-material absorber were simulated by CST Microwave Studio, as shown in Figure 7a. It can be found from the figure that, whether VO_2_ is in the di-electric state or in the metal state, the simulation results of FDTD Solutions and CST Microwave Studio are highly consistent. Furthermore, Figure 7b,c show the relative impedance of the absorber calculated by CST Microwave Studio. As mentioned in the impedance matching theory in the second part, when the effective impedance of the entire absorber is close to the impedance of free space, the absorber has very low reflection (high absorption) to the incident light. In this case, the real part of the relative impedance of the absorber is close to 1, and the imaginary part is close to 0. When comparing the spectrogram in Figure 7a with the relative impedance in Figure 7b,c, in the high absorption frequency band, the relative impedance satisfies the above conditions.

## 4. Conclusions

In summary, a bifunctional terahertz absorber based on a hybrid meta-material is proposed, which consists of a periodic array of gold resonators and VO_2_ resonators and a gold substrate separated by a TOPAS di-electric film. FDTD Solutions and CST Microwave Studio were used to simulate the data of the absorber. The simulation results show that with the switching of VO_2_ state, the designed meta-material absorber can achieve a reversible conversion between the ultra-wideband absorption and triple-band absorption in the terahertz band. When VO_2_ is in the metallic state, ultra-wideband absorption with a bandwidth of 6.62 THz, ranging from 4.71 THz to 11.33 THz, is achieved. In the case where VO_2_ is in the di-electric state, only the gold resonators participate in response to the incident light. Three absorption bands are achieved, with absorption frequencies and absorption intensities of 10.57 THz and 96.9%, 12.68 THz and 81.9%, and 13.91 THz and 91.9%, respectively. By utilizing the electromagnetic field distributions, the physical mechanisms of ultra-broadband absorption and triple-band absorption are discussed. Furthermore, the influence of structural parameters on absorption response is also discussed. The designed bifunctional meta-material absorber should have great application potential in the terahertz band for applications such as photocatalysis, photoelectric conversion, and electromagnetic stealth.

## Figures and Tables

**Table 1:** A comparison of similar types of meta-material absorbers in recent years.

Reference	Whether It Has Two Absorption Modes	Broadband Absorption	Multiband Absorption
		Bandwidth (Absorption Rate)	Number of Peak(s)
[49]	Yes	2.55 THz (>90.0%)	3
[50]	Yes	0.22 THz (>95.4%)	2
[51]	Yes	0.55 THz (>90.0%)	2
[52]	Yes	0.66 THz (>98.0%)	3
[53]	Yes	0.38 THz (>90.0%)	1
[54]	No	10.76 THz (>90.0%)	/
[55]	No	6.00 THz (>97.0%)	/
[56]	No	5.50 THz (>90.0%)	/
This paper	Yes	6.62 THz (>90.0%)	3

**Table 2:** Structural parameters of the designed terahertz meta-material absorber.

Parameter	*L*_1_	*L*_2_	*l*_1_	*l*_2_	*P*	*d*
Value (μm)	11	14	14	5	16	0.5
Parameter	*w*_1_	*w*_2_	*t*_1_	*t*_2_	*t*_3_	
Value (μm)	1	2	0.6	6	1	

## Data Availability

The data that support the findings of this study are available from the corresponding author upon reasonable request.
